# Delayed Partial Nephrectomy After Renal Cryoablation: Whole-Lesion Histology and Clinical Course of a Single Case

**DOI:** 10.3390/jcm15124479

**Published:** 2026-06-10

**Authors:** Alimire Maimaitijiang, Yaohui Wang, Zhaopei Liu, Qingzhi Xiang, Hui Zhu, Xuejun Zhang, Hualei Gan, Yu Zhu

**Affiliations:** 1 Department of Urology, Fudan University Shanghai Cancer Center, Shanghai 200032, China; 24211230008@m.fudan.edu.cn (A.M.);; 2 Department of Oncology, Shanghai Medical College, Fudan University, Shanghai 200032, China; 3 Department of Interventional Radiology, Fudan University Shanghai Cancer Center, Shanghai 200032, China; 4 Department of Nursing, Fudan University Shanghai Cancer Center, Shanghai 200032, China; 5 Department of Pathology, Fudan University Shanghai Cancer Center, Shanghai 200032, China

**Keywords:** renal cryoablation, clear cell renal carcinoma, delayed partial nephrectomy, case report

## Abstract

**Introduction**: Cryoablation is an established nephron-sparing option for small renal masses, particularly in patients unsuitable for surgery. However, definitive histopathological assessment post-ablation is limited due to the in situ nature of treatment. This report details a case of delayed partial nephrectomy after cryoablation, enabling comprehensive histopathological evaluation of long-term treatment effects. **Case presentation**: A 50-year-old man with uncontrolled hypertension, diabetes, and triple-vessel coronary disease presented with a 2.5 cm right renal mass. Cardiovascular instability deferred initial surgery. Following coronary intervention requiring anticoagulation, percutaneous cryoablation was performed using CT-guided 3D reconstruction for precise probe placement and ice-ball confirmation. After 388 days, laparoscopic partial nephrectomy was performed. Histopathology revealed a 1.9 cm clear cell renal cell carcinoma. Approximately one-third of tissue showed post-cryoablation changes. Three distinct zones were identified: viable carcinoma, coagulative necrosis with preserved glomerular outlines, and viable parenchyma. Serial follow-up over 2 years showed transient creatinine elevation normalizing by 3 months, with no recurrence or metastasis. **Conclusions**: This case provides rare whole-lesion histopathological assessment after renal cryoablation, illustrating heterogeneous long-term tissue response and supporting cryoablation as a disease-control or bridging strategy in medically high-risk patients.

## 1. Introduction

Renal cell carcinoma (RCC) is the most common solid lesion within the kidney and accounts for approximately 90% of all kidney malignancies [1]. In recent years, the widespread use of cross-sectional imaging has led to an increase in the incidental detection of T1a RCC, defined as a tumor measuring ≤4 cm in greatest dimension and limited to the kidney [2,3]. Current guidelines for managing clinical stage T1a RCC recommend nephron-sparing surgery (NSS) as the standard of care treatment when technically feasible [4,5,6]. However, a proportion of patients are not suitable candidates for surgery because of older age, significant comorbidities, or multifocal disease. For these individuals, minimally invasive ablative techniques, including cryoablation, have become established treatment options for small renal masses [7,8,9,10].

Cryoablation can achieve favorable short-term oncologic and functional outcomes in selected patients, but its in situ nature generally limits postoperative histopathological assessment of the ablation zone. Most available histologic data on renal cryoablation derive from animal models or from salvage surgery performed for suspected local failure, and long-term whole-lesion human specimens are rare. Here, we describe a medically high-risk patient who underwent percutaneous renal cryoablation as a temporizing, disease-control strategy before delayed partial nephrectomy. This treatment sequence allowed integrated analysis of three-dimensional (3D) CT findings and whole-lesion histopathology 388 days after ablation, providing rare information on long-term tissue response at the cryoablation site.

## 2. Case Presentation

A 50-year-old man with hypertension and diabetes mellitus was incidentally found to have a 2.5 × 2.1 cm solid renal mass in the mid–lower pole of the right kidney on routine computed tomography (CT). His hypertension had been challenging to control and required combination therapy with irbesartan, metoprolol, nifedipine, atorvastatin, and nateglinide. He was admitted for elective preoperative evaluation before a planned laparoscopic partial nephrectomy (PN).

In August 2022, the patient developed spontaneous chest tightness, precordial discomfort, and markedly elevated blood pressure (183/93 mmHg). Electrocardiography revealed ischemic myocardial changes, and his symptoms resolved with oxygen therapy and oral nifedipine. In view of these findings, preoperative anesthetic assessment identified cardiovascular instability, and the planned laparoscopic PN was deferred. The patient was referred for cardiologic evaluation.

In October 2022, coronary angiography at Zhongshan Hospital, Fudan University, confirmed triple-vessel coronary artery disease with 70% stenosis of the proximal left anterior descending artery, 70% stenosis of the distal left circumflex artery, and chronic occlusion of the distal right coronary artery with collateral circulation. Coronary balloon angioplasty with prolonged antithrombotic therapy was recommended. Because of the anticipated need for long-term antithrombotic therapy and the associated perioperative bleeding risk, immediate nephron-sparing surgery was not considered appropriate. During multidisciplinary counseling, active surveillance, percutaneous cryoablation, and delayed partial nephrectomy were all discussed as guideline-supported options for a cT1a renal mass. Although active surveillance was offered as a reasonable choice given the small tumor size (<3 cm) and his cardiovascular comorbidity, the patient declined a purely observational strategy because of substantial anxiety about tumor progression. He elected for percutaneous cryoablation as an interim disease-control measure, with delayed partial nephrectomy retained as a definitive option once his cardiovascular status had been optimized.

Pre-procedural non-contrast and contrast-enhanced abdominal CT confirmed the solid mass in the posteroinferior region of the right kidney (Figure 1A). Under CT guidance with three-dimensional reconstruction, a single percutaneous variable-size cryoprobe (CVA2400, Endocare, Inc., Irvine, CA, USA) was advanced into the center of the lesion. Two freeze–thaw cycles were performed, each consisting of a 10 min active freeze followed by a 5 min active thaw. Serial CT scans documented progressive ice-ball formation (Figure 1B), and 3D reconstructions showed that the ice ball appeared to encompass the tumor in all directions (Figure 2).

Following coronary intervention and medical optimization of his cardiovascular status, the patient returned to our center. Preoperative contrast-enhanced CT at approximately 12 months after cryoablation showed a persistent non-enhancing ablation zone without appreciable shrinkage or morphological progression. Despite the non-enhancing imaging appearance, the patient preferred definitive surgical removal rather than continued surveillance, and laparoscopic partial nephrectomy was performed in November 2023, 388 days after cryoablation. Intra-operatively, the delayed partial nephrectomy was technically more demanding than a primary partial nephrectomy because of perinephric/peritumoral adhesions and partial loss of normal tissue planes around the prior ablation zone. Nevertheless, the procedure was completed laparoscopically without major intraoperative complications. Operative time was 170 min, estimated blood loss was 50 mL, and warm ischemia time was 15 min. Gross examination of the excised specimen showed a well-circumscribed, tan-brown lesion with focal hemorrhage (Figure 3A,B). Postoperative pathological analysis confirmed a 1.9 × 1.8 × 1.5 cm clear cell renal cell carcinoma. Immunohistochemistry showed positivity for PAX-8, CD10, and CAIX, with a Ki-67 proliferation index of approximately 2%; CK7 and TFE3 were negative (Supplementary Figure S1). Notably, approximately one-third of the tissue displayed fibrosis and infarction, indicative of changes consistent with post-cryoablation effects on renal lesions, correlating with the patient’s treatment history (Figure 3C). Histological analysis revealed coagulative necrosis with preserved glomerular outlines (Figure 3E). The cryoablated margin is well demonstrated as three distinct layers: renal cell carcinoma, coagulative necrosis, and viable renal parenchyma (Figure 3D).

Postoperatively, the patient was enrolled in a structured, multidisciplinary surveillance program. Laboratory follow-up demonstrated a transient rise in serum creatinine and blood urea nitrogen (BUN) immediately after surgery, which returned to baseline by the 3-month visit; uric acid levels remained within normal limits throughout the observation period (Supplementary Figure S2). Serial contrast-enhanced CT was performed at 3, 6, 15, 18, 21, and 24 months after partial nephrectomy (Figure 4). These studies showed postoperative changes at the resection site without evidence of local recurrence, regional lymphadenopathy, or distant metastasis. At 24 months after partial nephrectomy, follow-up CT revealed a new right adrenal lesion. To further characterize this finding and to exclude metastatic disease, both 18F-FDG PET and 68Ga-CAIX PET/CT were performed. Neither modality demonstrated abnormal tracer uptake in the adrenal lesion, findings that were not suggestive of metastatic RCC (Supplementary Figure S3). The adrenal lesion is being monitored with ongoing imaging follow-up.

## 3. Discussion

Renal cryoablation has emerged as a widely accepted nephron-sparing alternative for selected patients with small renal masses. Through repeated freeze–thaw cycles, this minimally invasive technique selectively induces tumor cell death while preserving adjacent renal parenchyma. Since its clinical introduction in the mid-1990s, cryoablation has consistently demonstrated favorable outcomes in oncologic control and renal function preservation. Nevertheless, histopathological evidence on long-term tissue response after renal cryoablation remains limited, largely because the treated kidney is usually left in situ and whole-lesion specimens are rarely available following clinically uneventful ablation.

Early insights into post-cryoablation histologic evolution have predominantly come from animal models. In porcine kidneys, cryoablation produces a central zone of coagulative necrosis that progresses over time to fibrosis, with early hemorrhage and subsequent hemosiderin deposition ultimately resulting in a well-demarcated, non-enhancing fibrotic scar [11]. Canine studies have shown that multiple freeze–thaw cycles yield more extensive tissue necrosis and more pronounced inflammatory remodeling than single-cycle protocols, underscoring the importance of technical optimization [12]. Other experimental work has consistently described a reproducible trajectory from acute inflammatory necrosis to chronic fibrotic scarring [13,14].

Human data remain comparatively sparse. Edmunds et al. reported on one of the first acute-phase histologic assessments, demonstrating extensive coagulative necrosis with no viable tumor cells in renal masses resected immediately after cryoablation [15]. Jang et al. later examined three patients who underwent laparoscopic cryoablation followed by delayed radical nephrectomy at a mean interval of approximately 275 days; their specimens showed central coagulative necrosis with macrophage-mediated debris clearance and no residual carcinoma, supporting sustained oncologic effect at an intermediate time point [16]. Longer-term (>1 year) histopathology has mainly been described in the context of salvage surgery for suspected local failure. In a multi-institutional series of 14 patients undergoing such surgery, Karam et al. found residual or recurrent RCC in most cases, although one specimen showed only fibrosis without tumor, illustrating that imaging suspicion may occasionally represent a false positive [17]. Additional reports have described late recurrences requiring further intervention, but detailed long-term correlations between imaging and whole-lesion histology remain uncommon, particularly in patients without overt clinical failure [18].

Against this background, the present case provides large-section, whole-lesion histology obtained 388 days after percutaneous renal cryoablation in a medically high-risk patient who initially underwent ablation as an interim tumor-control strategy. The resected specimen showed a three-zone architecture: a central area of viable clear cell RCC, a circumferential ring of coagulative necrosis with fibrotic change and preserved glomerular outlines, and an outer zone of viable renal parenchyma, with post-cryoablation fibrosis and infarction involving approximately one-third of the lesion. This configuration indicates the zonal heterogeneity of treatment effect and incomplete ablation, despite contemporary CT-guided planning, 3D reconstruction, and intra-procedural documentation of an ice ball that appeared to encompass the lesion. In contrast to many experimental and clinical descriptions that emphasize central necrosis with potential peripheral residual disease, our case demonstrates that the spatial distribution of viable tumor and necrosis can be more complex than anticipated from geometric ice-ball coverage alone.

The heterogeneous three-zone histology in this case may reflect the biological complexity of cryoablation injury. Tumor cell death after cryoablation results from ice-crystal formation, osmotic stress, membrane disruption, microvascular thrombosis, and ischemic injury during thawing [19]. Cryoablation may also trigger local inflammatory and immunogenic responses through the release of tumor antigens and damage-associated signals [20].

An additional feature of this case is the discrepancy between serial imaging and histology. During the interval between cryoablation and delayed partial nephrectomy, contrast-enhanced CT at 12 months showed a non-enhancing ablation zone without signs of local progression, and subsequent imaging after surgery has not identified recurrent or metastatic RCC to date. Whole-lesion histology, however, revealed a central focus of viable carcinoma within the previously treated mass. This observation suggests that, at least in some patients, imaging appearances consistent with complete ablation may not fully reflect the underlying histologic status of the ablation zone. Rather than undermining the role of imaging, this highlights the need to interpret post-ablation studies within the known limitations of current modalities and to maintain structured, long-term surveillance, particularly in higher-risk settings. Building on the biological mechanisms outlined above, several non-mutually exclusive factors may underlie this imaging–histology mismatch. First, the visible ice-ball margin does not precisely correspond to the lethal isotherm, and peritumoral vascular perfusion can create a local heat-sink effect that protects tumor cells near large intrarenal vessels. In addition, post-ablation necrotic or fibrotic remodeling may obscure small foci of viable tumor on routine contrast-enhanced CT [19,21]. These observations support cautious interpretation of non-enhancement as a surrogate for complete histologic eradication.

From a clinical-management perspective, this imaging–histology mismatch raises the practical question of which patients are most at risk of incomplete ablation and how such failures might be detected earlier. Reported risk factors include larger tumor size, central or hilar location, endophytic growth pattern, proximity to large intrarenal vessels with perfusion-mediated tissue warming, inadequate ablative margins beyond the visible tumor edge, suboptimal probe configuration, and variability in real-time ice-ball monitoring [10,21]. Potential strategies to reduce or identify incomplete ablation include standardized pre-procedural 3D planning, use of multi-probe arrays for selected tumors, intra-procedural assessment of the ablative margin, structured contrast-enhanced imaging surveillance, and selective post-ablation biopsy in higher-risk or equivocal lesions [10,22]. In selected cases, complementary functional imaging, including 18F-FDG PET or CAIX-targeted PET, may help characterize indeterminate findings that conventional contrast-enhanced CT alone cannot resolve [23].

The clinical trajectory in this patient also illustrates how comprehensive surveillance can inform the evaluation of incidental findings on follow-up imaging. At 24 months after partial nephrectomy, a new right adrenal lesion was detected on CT. Dual-tracer assessment with 18F-FDG PET and CAIX-PET showed no abnormal uptake, making metastatic RCC unlikely, and the lesion is being monitored.

More broadly, this case underscores the role of cryoablation in the contemporary management of small renal masses. Because of triple-vessel coronary artery disease and the need for prolonged antithrombotic therapy, immediate nephron-sparing surgery was deferred and the patient elected percutaneous cryoablation after detailed counseling. Ablation provided local disease control during a period of high surgical risk and preserved renal function. Once his cardiovascular status had been optimized, delayed partial nephrectomy removed the residual mass with negative margins and provided definitive histologic assessment of the ablation bed. In this setting, cryoablation functioned as a temporizing, disease-control measure before delayed definitive surgery. In addition, subsequent partial nephrectomy after cryoablation may be technically more demanding because of adhesions and altered tissue planes, which should be considered during shared decision-making when ablation is selected as a bridging or temporizing strategy.

This report has several limitations. It describes a single patient, limiting generalizability. No pre-ablation biopsy, interval biopsy, or germline/somatic molecular testing was performed, which limits baseline tumor characterization, determination of the timing of residual viability, and biological interpretation. In addition, although 24 months of post-surgical surveillance are reported, longer follow-up is required to assess durable oncologic control. Finally, the findings reflect a single-center experience with a specific cryoablation and surveillance approach.

## 4. Conclusions

In summary, this case provides whole-lesion histology of a clear cell renal cell carcinoma 388 days after percutaneous cryoablation, performed as a temporizing strategy in a medically high-risk patient. Despite serial contrast-enhanced CT consistent with complete ablation, large-section histology demonstrated a central focus of viable carcinoma surrounded by zones of coagulative necrosis and viable parenchyma.

## Figures and Tables

**Figure 1:**
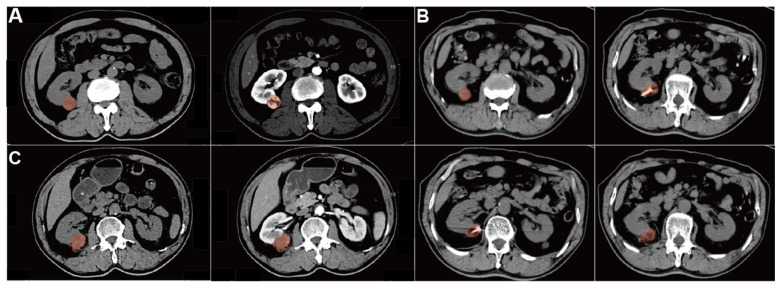
Axial CT images of the right renal mass and ablation zone. (**A**) Baseline unenhanced and contrast-enhanced CT showing a 2.5 × 2.1 cm enhancing mass in the mid–lower pole of the right kidney. (**B**) Intra-procedural CT with a single percutaneous cryoprobe in situ and an ice ball surrounding the lesion. (**C**) Post-cryoablation CT showing a hypoattenuating, non-enhancing ablation zone at the treatment site. CT, computed tomography.

**Figure 2:**
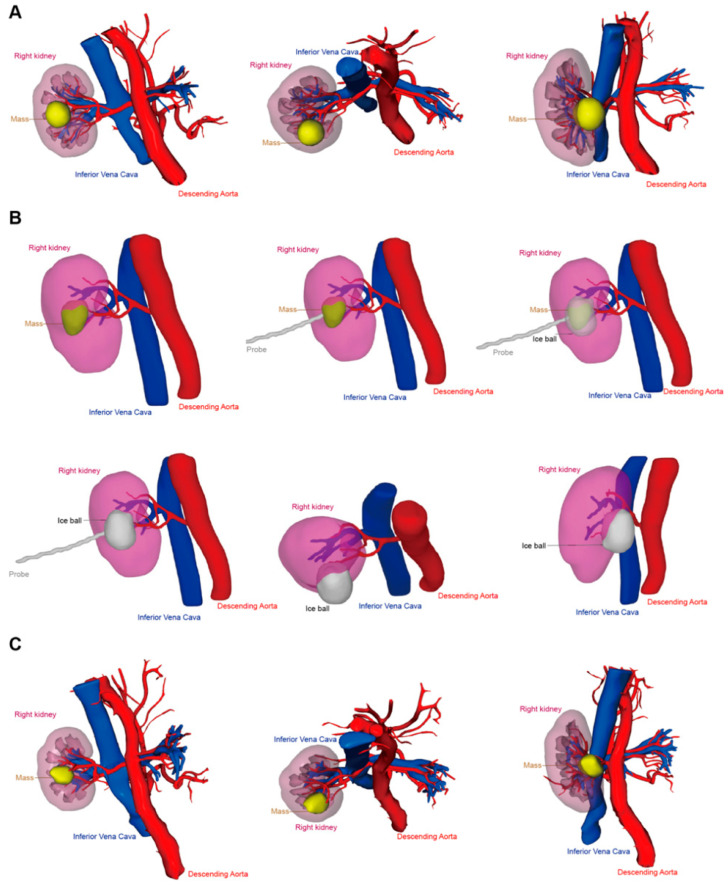
Three-dimensional CT reconstructions before and during cryoablation, and before partial nephrectomy. (**A**) Pre-cryoablation 3D reconstruction demonstrating the right kidney, renal mass (yellow), aorta (red) and inferior vena cava (blue). (**B**) Intra-procedural 3D views showing the cryoprobe and the ice ball (gray) apparently encompassing the tumor. (**C**) Pre-partial nephrectomy 3D reconstruction indicating the residual mass and its relation to major vessels, used for surgical planning.

**Figure 3:**
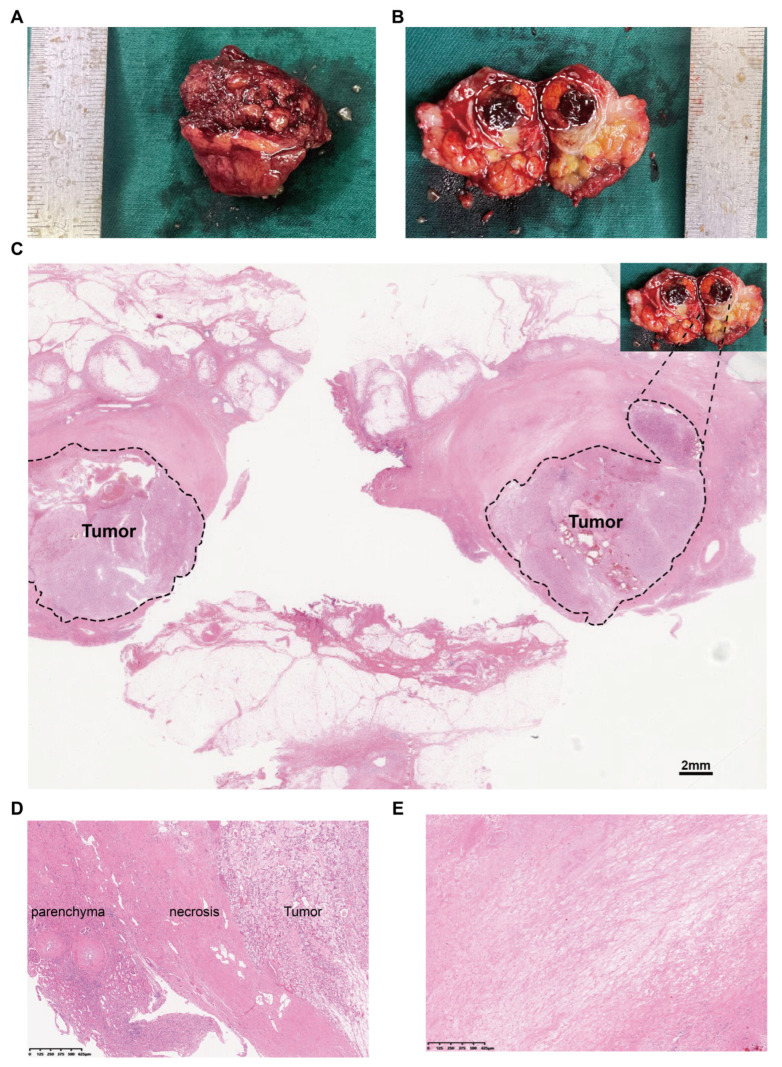
Gross and histopathological findings after delayed partial nephrectomy. (**A**,**B**) Gross photographs show a well-circumscribed, tan-brown, focally hemorrhagic post-cryoablation lesion; in (**B**), the white dashed lines outline the gross lesion areas on the cut surface. (**C**) Hematoxylin and eosin (H&E) section demonstrating fibrosis and infarction consistent with post-cryoablation changes; the focal tissue discontinuity in (**C**) is related to large-section preparation and does not obscure the pathological findings shown in this section. (**D**) From left to right, renal cell carcinoma, coagulative necrosis, and viable renal parenchyma are shown (reduced from 4× magnification). (**E**) Coagulative necrosis with glomerular outlines (reduced from 4× magnification).

**Figure 4:**
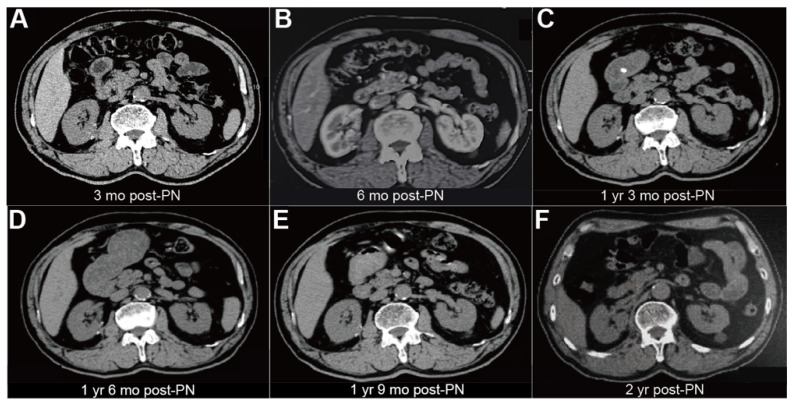
Serial CT after cryoablation and delayed partial nephrectomy. Axial CT at 3, 6, 15, 18, 21 and 24 months after partial nephrectomy (**A**–**F**) showing the stable postoperative appearance of the right kidney without local recurrence. At 24 months (**F**), a new right adrenal lesion is visible and was subsequently evaluated with PET/CT. CT, computed tomography; PET, positron emission tomography; mo, month; yr, year.

## Data Availability

The original contributions presented in this study are included in the article and Supplementary Materials. Further inquiries can be directed to the corresponding authors. High-resolution pathological images are available from the corresponding authors upon reasonable request.

## Supplementary Materials

The following supporting information can be downloaded at https://www.mdpi.com/article/10.3390/jcm15124479/s1: Figure S1: Immunohistochemical staining of normal renal tissue and tumor tissue for CAIX, PAX8, CK7, Ki-67, CD10 and TFE3; Figure S2: Serial measurements of serum creatinine, blood urea nitrogen and uric acid from initial diagnosis to 12 months after partial nephrectomy; Figure S3: PET/CT evaluation of the right adrenal lesion 24 months after partial nephrectomy.

## Abbreviations

The following abbreviations are used in this manuscript:

3D-CT	three-dimensional computed tomography
BUN	blood urea nitrogen
CT	computed tomography
H&E	hematoxylin and eosin
PN	partial nephrectomy
RCC	renal cell carcinoma
