# Supplementary material for: Delayed Partial Nephrectomy After Renal Cryoablation: Whole-Lesion Histology and Clinical Course of a Single Case
Source: J Clin Med. 2026 Jun 10;15(12):4479. doi: 10.3390/jcm15124479 (PMC13302037; doi:10.3390/jcm15124479)
Supplement: Supplementary file 1 [file jcm-15-04479-s001.zip › jcm-4274861-supplementary.pdf]

## Supplementary Materials：

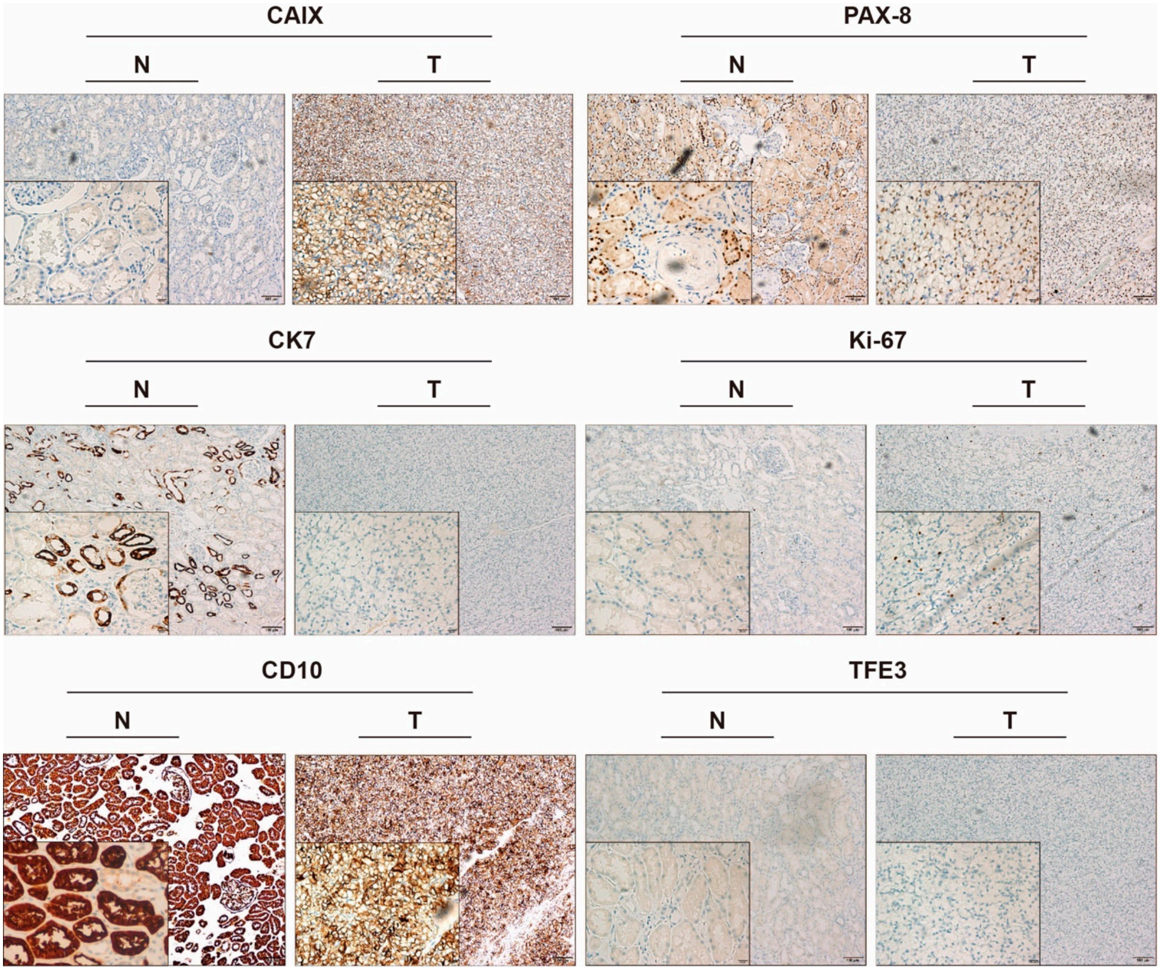


**Supplementary Figure S1.** For each marker (CAIX, PAX8, CK7, Ki-67, CD10 and TFE3), the left panel shows normal renal tissue and the right panel shows tumor tissue. Main panels correspond to 10× magnification fields; insets show the corresponding 40× high-power views. Scale bars: main panels, 100 μm; insets, 20 μm.

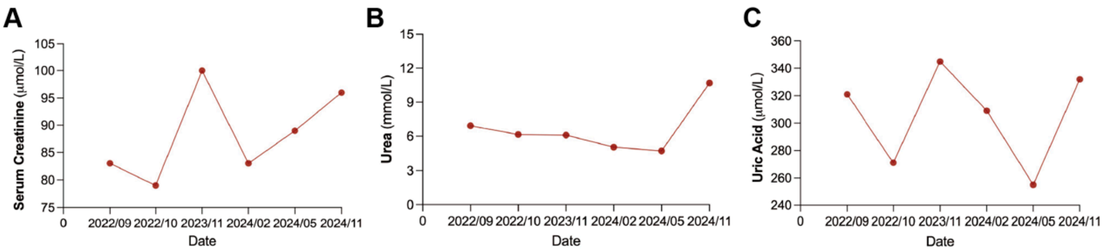


**Supplementary Figure S2.** Serial measurements of **(A)** serum creatinine, **(B)** blood urea nitrogen and **(C)** uric acid from initial diagnosis to 12 months after partial nephrectomy.

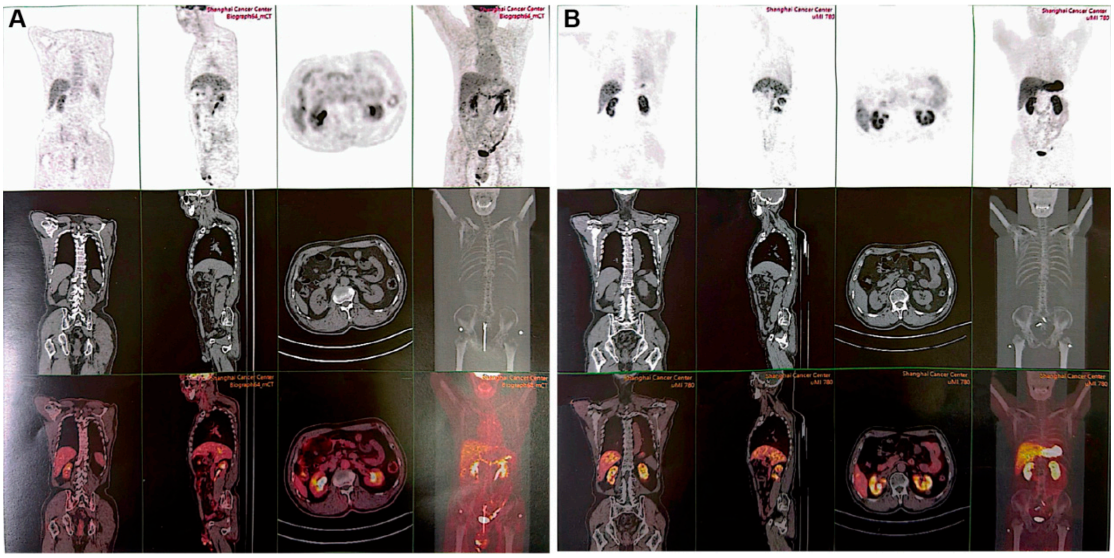


**Supplementary Figure S3.** PET/CT evaluation of the right adrenal lesion 24 months after partial nephrectomy. (**A**) $^{18}$F-FDG PET/CT fused image showing no abnormal FDG uptake in the right adrenal lesion. (**B**) CAIX-PET/CT fused image likewise demonstrating no increased tracer uptake, findings not suggestive of metastatic renal cell carcinoma. FDG, fluorodeoxyglucose.
